# Gene expression changes contribute to stemness and therapy resistance of relapsed acute myeloid leukemia: roles of *SOCS2, CALCRL, MTSS1*, and *KDM6A*

**DOI:** 10.1016/j.exphem.2021.05.004

**Published:** 2021-05-21

**Authors:** Alexander M. Grandits, Rotraud Wieser

**Affiliations:** a Division of Oncology, Department of Medicine I, Medical University of Vienna, Vienna, Austria; b Comprehensive Cancer Center, Vienna, Austria

## Abstract

Relapse is associated with therapy resistance and is a major cause of death in acute myeloid leukemia (AML). It is thought to result from the accretion of therapy-refractory leukemic stem cells. Genetic and transcriptional changes that are recurrently gained at relapse are likely to contribute to the increased stemness and decreased therapy responsiveness at this disease stage. Despite the recent approval of several targeted drugs, chemotherapy with cytosine arabinoside and anthracyclines is still the mainstay of AML therapy. Accordingly, a number of studies have investigated genetic and gene expression changes between diagnosis and relapse of patients subjected to such treatment. Genetic alterations recurrently acquired at relapse were identified, but were restricted to small proportions of patients, and their functional characterization is still largely pending. In contrast, the expression of a substantial number of genes was altered consistently between diagnosis and recurrence of AML. Recent studies corroborated the roles of the upregulation of *SOCS2* and *CALCRL* and of the downregulation of *MTSS1* and *KDM6A* in therapy resistance and/or stemness of AML. These findings spur the assumption that functional investigations of genes consistently altered at recurrence of AML have the potential to promote the development of novel targeted drugs that may help to improve the outcome of this currently often fatal disease.

In acute myeloid leukemia (AML), excessive proliferation of partially differentiated or undifferentiated blasts supersedes the production of normal blood cells, thereby causing infections, anemia, bleeding, and, in untreated patients, death within months. The annual incidence of this aggressive hematopoietic malignancy is ~5/100,000, and the median age at diagnosis ~70 years [1−3]. Similar to normal hematopoiesis, leukemic hematopoiesis is organized in a hierarchical manner; that is, the highly proliferative bulk leukemic cells (LCs) are derived from mostly quiescent leukemic stem cells (LSCs), which reside in a specialized stem cell niche in the bone marrow (BM) [4−6]. Although not undisputed, LSCs are considered to represent a reservoir of therapy resistance and the source of relapse [4−6].

Like other malignant diseases, AML is caused by genetic and molecular alterations. These occur in hematopoietic stem or progenitor cells, transforming them into LSCs [5,7], and include cytogenetic aberrations, copy-number variations, small insertions/deletions, point mutations, and epigenetic and transcriptional changes [8−12]. An array of different recurrent genetic alterations has been described in AML, leading to substantial genetic heterogeneity between patients. These recurrent aberrations may act as drivers of leukemogenesis and serve as prognostic markers and/or as targets for rationally designed therapies [3,8−11,13].

The first molecularly targeted drug in AML was *all-trans*-retinoic acid, which, since the late 1980s, has greatly improved the outcome of patients with rearrangements of the retinoic acid receptor a gene [14]. In the past few years, additional targeted therapeutics, including tyrosine kinase inhibitors, BCL2 inhibitors, isocitrate dehydrogenase (IDH) inhibitors, and antibody −drug conjugates, have been approved for certain subgroups of AML [3,13]. Nevertheless, chemotherapy remains the first choice for fit patients with AML; moreover, targeted drugs are often used in combination with it [3,13]. Standard AML chemotherapy consists of cytosine arabinoside (araC) and an anthracycline (e.g., daunorubicin or idarubicin), and is complemented by hematopoietic stem cell (HSC) transplantation in select cases [3]. However, 5-year survival ranges only between <5% and ~40%, depending on prognostic parameters such as age, white blood cell count, and the presence of specific genetic and gene expression alterations [1−3,9]. This poor outcome is to a large extent due to the fact that, even though a majority of patients achieve complete remission, many of these patients relapse with largely therapy-resistant disease [3,15].

## Genetic and transcriptional changes between diagnosis and relapse of AML

One plausible hypothesis explaining therapy resistance at relapse is that it is caused by genetic, epigenetic, and/or transcriptional alterations newly appearing at this disease stage [12]. These changes may already have been present in a fraction of the usually clonally heterogeneous leukemic cell population at diagnosis, or they may have newly emerged during, or even as a consequence of, treatment. In either case, their presence in a major proportion of leukemic cells at relapse is likely to reflect a survival advantage under the selective pressure associated with therapy [12]. Accordingly, a number of studies have compared cytogenetic aberrations, copy-number variations, uniparental isodisomies (UPDs), small insertions/deletions, and point mutations between diagnosis and relapse of AML. We recently comprehensively compiled these studies with the intention to identify potential drivers of relapse fulfilling the following criteria: being recurrently gained or strongly increased in abundance at relapse, not being lost/ strongly decreased in abundance at relapse in other patients, and not recurrently occurring at diagnosis, or being associated with poor therapy responsiveness if present at this stage [12]. Candidate lesions fulfilling

these criteria were deletions of the long arms of chromosomes 5 and 7, UPD(13q) (leading to homozygosity of *FLT3* internal tandem duplications already present in a heterozygous state at diagnosis), and deletions or mutations of the tumor suppressor gene TP5*3*. All of these changes were found only in low single-digit percentages of patients [12], potentially indicating that the genetic basis of therapy resistance at relapse is as heterogeneous as the genetic makeup of AML at diagnosis. Along these lines, in a recently published analysis, several genes that recurrently gained mutations at relapse were newly identified through whole-genome sequencing of a relatively large number of diagnosis and relapse samples, including 27 paired samples from adult AML patients [16]. Mutations in each of these genes were acquired in a small number of patients. They had probably escaped detection in previous analyses because most of the affected genes were not part of AML-specific sequencing panels, and whole-genome sequencing had been applied only to small numbers of samples [16]. These mutations await confirmation of their relapse-specific nature in independent patient cohorts, as well as, in most cases, elucidation of their contributions to relapse-associated features. Other recent developments concern the investigation of mutational patterns at diagnosis and relapse of various cytogenetically or genetically determined AML subgroups [17], clonal evolution under targeted therapy [18,19], and the application of single-cell sequencing to the investigation of the evolution of AML from diagnosis to relapse [20].

A related line of research addressed the potential contribution of epigenetic and gene expression changes to relapse-related disease properties. The methylation density of the CpG islands (CGIs) located in the regulatory regions of certain preselected genes was significantly increased in relapsed versus diagnostic AML samples [21]. Furthermore, a genomewide gene expression analysis on paired samples from 11 patients with a normal karyotype at diagnosis uncovered 536 and 551 genes that were significantly up- and downregulated at relapse, respectively [22]. Despite the relatively small number of samples included in this comparison, downstream analyses supported the validity of the resulting relapse-associated gene expression signature. Thus, this signature was enriched for previously identified gene expression signatures associated with AML LSCs and/ or with poor outcome of AML [22], supporting the notion that specific gene expression patterns contribute to stemness and therapy resistance of both newly diagnosed and relapsed AML. Indeed, several of the genes that were upregulated at relapse had been previously implicated in these or related features, for example, *ERG* [23], *MYCN* [24], *MSI2* [25], WT1 [26,27], *DNMT3B* [28], *ADGRG1* (alias *GPR56*) [29], and *PRDM16* [30]. Additionally, some genes in the relapse signature with a previously largely uncharacterized role in AML were selected for functional characterization and found to contribute to AML stemness and/or therapy resistance as outlined below.

### Suppressor of cytokine signaling 2 (SOCS2)

Several gene expression signatures associated with poor outcome in AML have been established, but most of these were either based on preselected genes and/or contained too many genes to be readily applicable to clinical practice [11,31−38]. We therefore employed an unbiased approach to establish a prognostic signature consisting of a small number of genes [39]. The resulting signature comprised four genes: *SOCS2*, *IL2RA, NPDC1*, and *PHGDH*. Its elevated expression was an independent prognostic parameter for poor overall survival in several independent AML cohorts [39]. Interestingly, *SOCS2* and *NPDC1* were also upregulated at relapse of AML [22], supporting their potential contribution to AML progression and therapy resistance. Such a role was experimentally probed for *SOCS2*, a component of the JAK−STAT signaling pathway. This pathway is aberrantly activated in several tumor types [40]. In AML, it plays a key leukemogenic role and enhances the growth and maintenance of LSCs [41]. *SOCS* genes are transcriptionally induced by JAK−STAT signaling [40]. They encode substraterecruiting components of E3−ubiquitin ligase complexes, which initiate the degradation of cytokine receptors and signaling proteins, thus acting as negative feedback regulators of the pathways leading to their induction [40]. Based on this function, they may be expected to act as tumor suppressors; correspondingly, *SOCS2* was downregulated in several cancer types [40]. On the other hand, elevated SOCS2 levels were found in colon and prostate cancer and were associated with a poor prognosis in the latter [42,43]. Moreover, *SOCS2* promoted proliferation, anchorage-independent growth, apoptosis resistance, and in vivo tumor growth of prostate and colon cancer cell lines [42−44], testifying to its oncogenic potential.

In the healthy murine hematopoietic system, *Socs2* was highly expressed in HSCs and required for stress hematopoiesis [45]. *SOCS2* expression was significantly increased in patients with chronic myeloid leukemia (CML) in blast crisis as compared with chronic phase patients and healthy controls [46]. In AML, a crucial role for JAK−STAT signaling is well documented [41], and *SOCS2* was upregulated as compared with healthy controls [47], yet little was known about the functional role of *SOCS2* in this disease.

**Figure 1:**
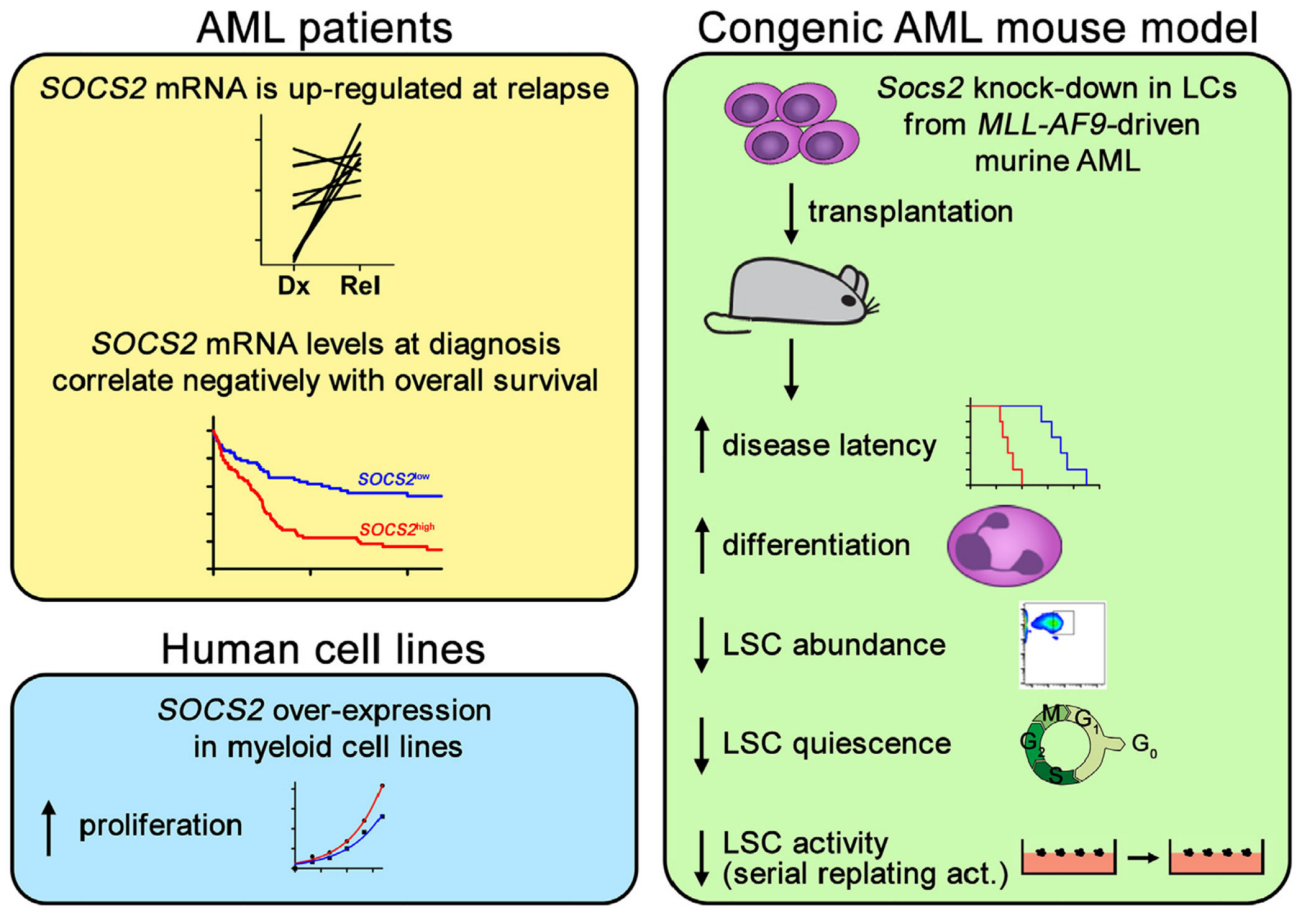
Upregulation of *SOCS2* is associated with relapse and poor outcome of AML, and *SOCS2* promotes AML progression and stem cell-related properties. *act*=activity; *Dx*=diagnosis; *Rel*=relapse.

In our studies, experimental expression of *SOCS2* enhanced the proliferation of the malignant human myeloid cell lines U-937 and HL-60 [39] (Figure 1). Furthermore, expression of *Socs2* was strongly elevated in LCs from both an *Flt3*-ITD/*Npm1c-* and an *MLL-AF9*-driven mouse model of AML as compared with normal murine hematopoietic cells [39]. Attempts to knock down *Socs2* in *Flt3*-ITD/*Npm1c*-driven murine AML using short hairpin RNAs (shRNAs) led to a rapid and complete loss of shSocs2-, but not of shCtrltransduced LCs in culture. Even though this precluded the use of these cells for downstream experiments, it impressively illustrated an essential role of *Socs2* in the proliferation and/or survival of *Flt3*-ITD/*Npm1c*-driven LCs [39]. In the *MLL-AF9* model, knockdown of *Socs2* also inhibited LC proliferation in vitro, yet sufficient cell numbers for transplantation experiments could be recovered. SOCS2 depletion increased disease latency and promoted the myeloid differentiation of LCs in vivo. Moreover, it reduced the abundance and quiescence of immunophenotypically defined LSCs, as well as LSC activity as determined through a serial replating assay [39] (Figure 1).

In summary, expression and functional data support a role for *SOCS2* in AML aggressiveness and stemness, possibly reflecting a role not only as a negative regulator of the JAK−STAT pathway, but also as a down-stream target of it.

### Calcitonin receptor-like receptor (CALCRL)

In parallel to our own above described effort, Wagner et al. [48], using a machine-learning approach, established a three-gene signature with prognostic significance in several independent AML data sets [48]. The top gene in this signature was *CALCRL*, which also ranked among the top differentially expressed genes between diagnosis and relapse of AML [22]. Both the prognostic significance of CALCRL mRNA and protein expression at diagnosis [49,50] and the upregulation of *CALCRL* at relapse [48] were confirmed independently and suggest a possible role for this gene in both primary and relapse-associated therapy resistance.

*CALCRL* encodes a G-protein-coupled seven-transmembrane domain receptor, which requires one of three single transmembrane domain co-receptors, RAMP1, RAMP2, or RAMP3, for cell surface expression and binding of its peptide ligands [51]. The ligand for the CALCRL/RAMP1 complex is calcitonin generelated peptide (CGRP), whereas adrenomedullin (ADM) binds to CALCRL/RAMP2 and CALCRL/ RAMP3 complexes [51−53]. CGRP and ADM have multiple physiological roles, including the regulation of blood pressure [52−56]. They also contribute to various pathologic processes [53,56], and the key role of CGRP in migraines has led to the development and regulatory approval of inhibitory antibodies and small molecule antagonists [55]. On the basis of gene expression data [52,56−60] and their abilities to stimulate proliferation, migration, invasiveness, and angiogenesis and to inhibit apoptosis and antitumor immune responses [52,56,57,59−65], CALCRL and its ligands have also been implicated in the pathogenesis of various malignant diseases. Accordingly, genetic or pharmacologic inhibition of CGRP or ADM signaling reduced tumor-related properties in vitro and in animal models [52,56,57,59,65].

ADM was expressed in hematopoietic cells [60,66,67] and was strongly induced by hypoxia [60,67], a condition thought to characterize the HSC niche in the BM [68]. Functions of CGRP signaling in the hematopoietic system were suggested by the abundant presence of CGRP immunoreactive nerve fibers in BM [54,69,70] and the expression of CALCRL and RAMP1 on hematopoietic cells [70−72]. CGRP stimulated proliferation and inhibited apoptosis of hematopoietic cells in vitro [62,73]. In vivo, targeted deletion of *Ramp1*, the only *Ramp* gene expressed in the HSC-enriched murine Lin^−^Sca-1^+^Kit^+^ (LSK) population, impaired hematopoiesis under various stress conditions [72]. Most recently, BM nociceptor-derived CGRP was reported to act directly on murine HSCs to promote their egress from the BM [70].

**Figure 2:**
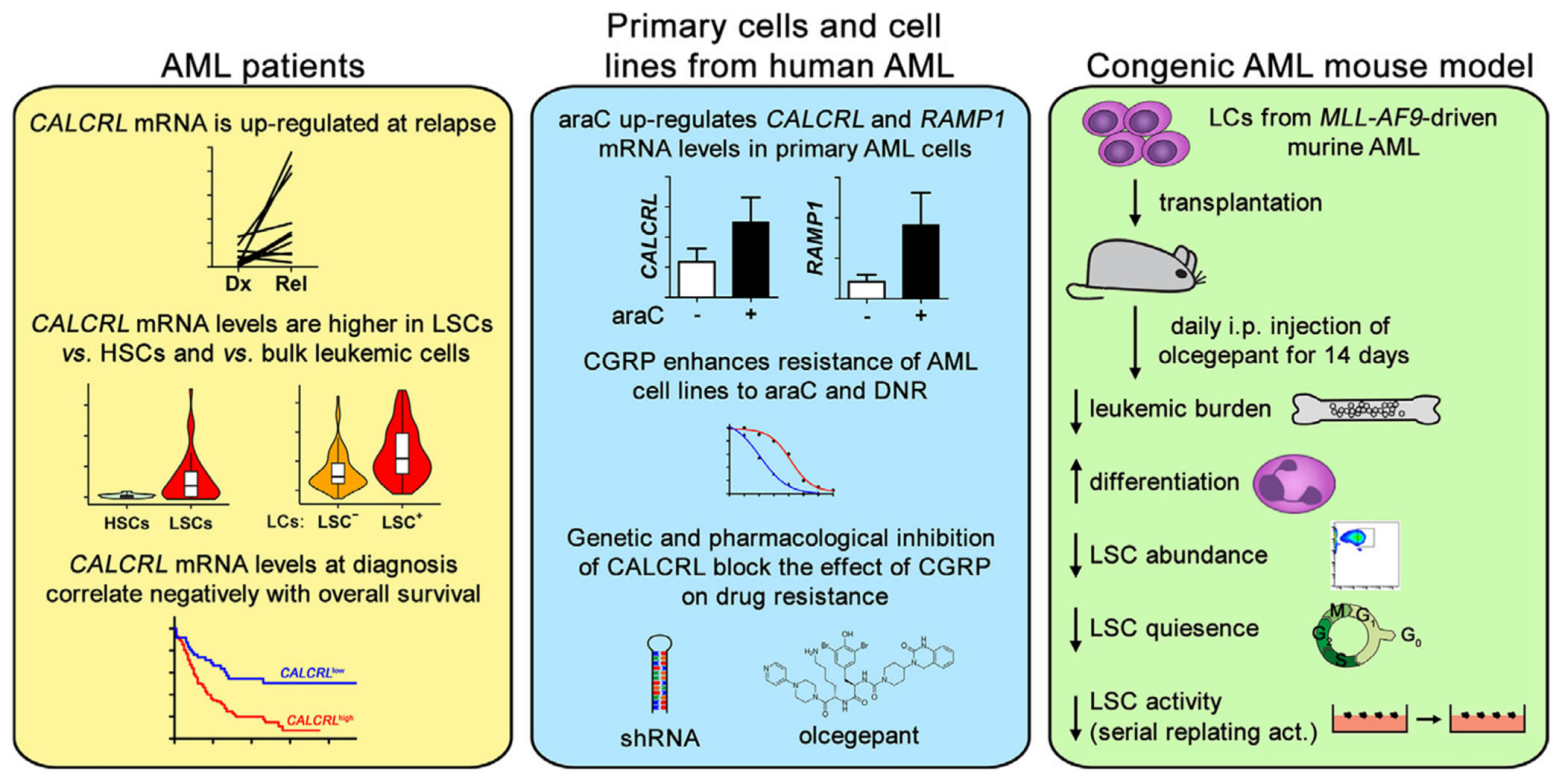
Upregulation of *CALCRL* is associated with relapse, leukemic stem cells, and poor outcome of AML, and CGRP signaling via CALCRL promotes therapy resistance and stem cell−related properties in AML. *act*=activity; *DNR*=daunorubicin; *Dx*=diagnosis; *i.p.*=intraperitoneal; *Rel*=relapse.

Given the association between high *CALCRL* expression and therapy resistance in patients with AML, and the functions of *CALCRL* in normal HSCs as well as in other malignancies, we set out to investigate a possible role for *CALCRL* in AML. Quantitative reverse transcription polymerase chain reaction (qRT-PCR) confirmed the expression, and the upregulation at relapse, of *CALCRL* in primary human AML cells. Interestingly, among the *RAMP* genes, only *RAMP1* mRNA was detectable in all analyzed specimens, whereas *RAMP2* and *RAMP3* transcripts were measurable only in 0 and 25% of the samples, respectively, suggesting that CALCRL acts mainly as a CGRP, rather than as an ADM, receptor in AML [74]. Treatment of primary AML cells with araC led to further transcriptional induction of *CALCRL* and *RAMP1* [74] (Figure 2). In publicly available genomewide gene expression data, *CALCRL* was upregulated in LSC-enriched versus LSC-depleted AML cell populations and in LSC-versus HSC-enriched cell populations and was an independent prognostic parameter for poor outcome in several AML cohorts [74] (Figure 2). CGRP enhanced the resistance of two *CALCRL-* and *RAMP1*-expressing human AML cell lines to araC and daunorubicin, and this effect was counteracted both by shRNA-mediated downregulation of *CALCRL* and by receptor inhibition through the truncated peptide, CGRP_(8−37)_, or the small molecule olcegepant [74] (Figure 2). In an *MLL-AF9*-driven congenic AML mouse model, in vivo treatment with olcegepant reduced leukemic burden in BM and spleen, promoted myeloid differentiation, reduced the abundance and quiescence of an immuno-phenotypically defined LSC-enriched cell population [74], and decreased serial replating ability as a measure of LSC activity (AMG, unpublished results) (Figure 2). In summary, these data indicate that CGRP−CALCRL signaling promotes chemotherapy resistance and stem cell-related properties in AML. Although constitutive deletion of *Ramp1* impeded stress hematopoiesis in mice [72], recent results indicate that antagonizing CGRP might even positively affect the numbers of normal HSCs in BM [70]. Also, combined treatment with araC and olcegepant significantly increased the proportion of murine LSK cells in patient-derived xenograft (PDX) models [50]. Thus, CGRP−CALCRL inhibition could potentially provide dual benefit in AML by helping to eliminate AML cells and additionally augmenting normal BM HSCs.

**Figure 3:**
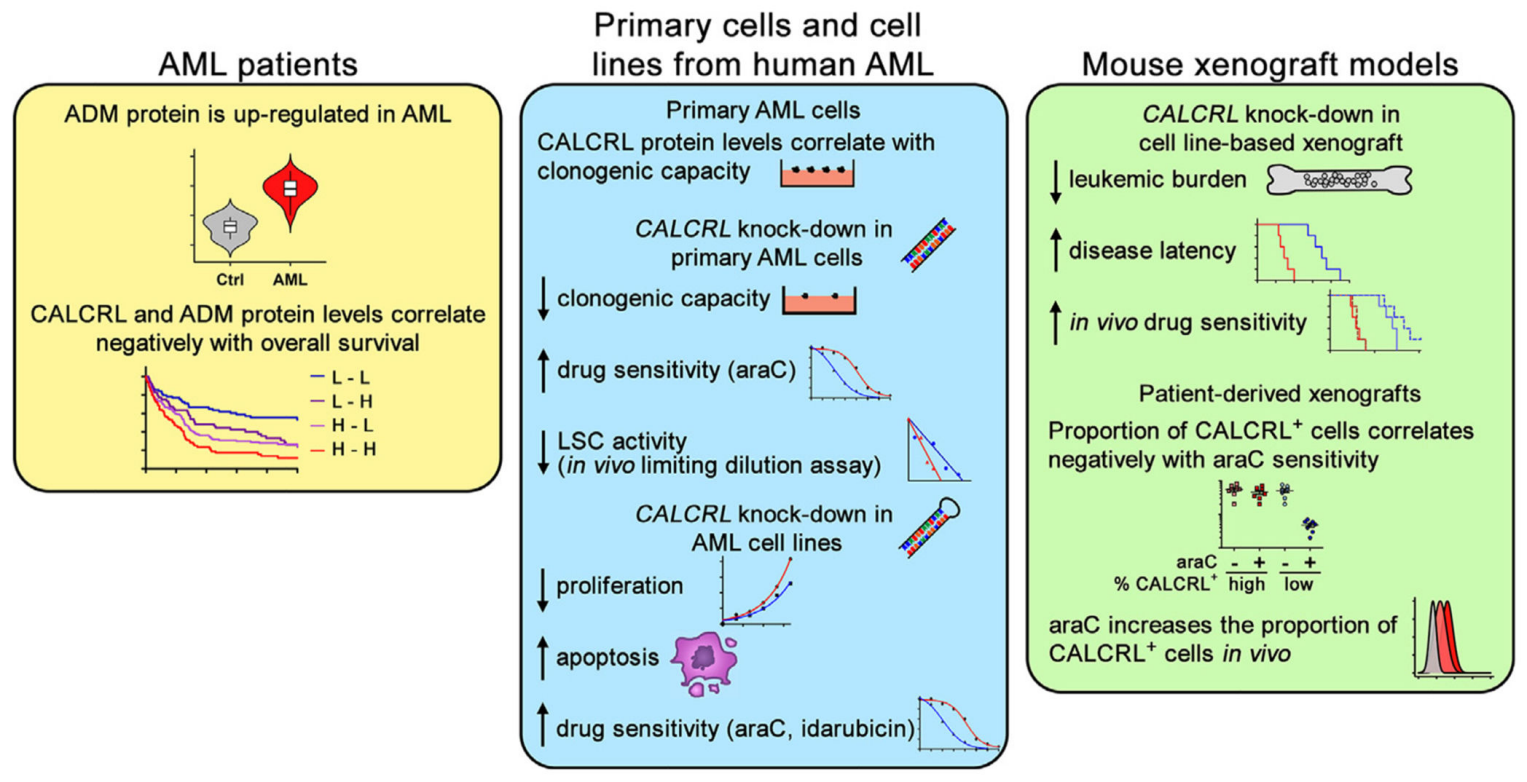
Upregulation of ADM and CALCRL is associated with poor outcome of AML, and *CALCRL* promotes AML stemness and therapy resistance. *Ctrl*=control; *H*=high; *L*=low.

Most recently, Larrue et al. [50] confirmed the pivotal role of *CALCRL* in AML [50]. They showed that knockdown of *CALCRL* in human AML cell lines (that were different from the ones we had used) decreased proliferation, increased apoptosis, and enhanced cellular sensitivity to araC and idarubicin (Figure 3). In cell line−based mouse xenograft models, *CALCRL* knockdown led to a reduction of leukemic burden, prolonged animal survival, and enhanced the effects of araC treatment [50]. In primary AML cells, CALCRL protein levels correlated with clonogenic capacity in methyl cellulose. siRNA-mediated CALCRL depletion decreased clonogenic capacity, increased cellular sensitivity to araC, and decreased LSC frequency as measured through an in vivo limited dilution assay [50] (Figure 3). In PDX, a higher proportion of CALCRL-positive cells was associated with reduced araC responsiveness. Both in PDX and in human patients, in vivo treatment with araC or araC-containing chemotherapy, respectively, increased the proportion of CALCRL-positive cells [50] (Figure 3). In agreement with our own findings [74], these experiments pointed toward a pivotal role for *CALCRL* in AML stemness and therapy resistance. However, Larrue et al. [50] reached different conclusions regarding the relevant ligand. They reported that ADM was overexpressed in AML versus control cells and that high ADM and CALCRL protein levels were both associated with poor outcome in AML (Figure 3). Knockdown of *ADM* or *RAMP2* mimicked some of the effects of the *CALCRL* knockdown. Finally, in PDX, olcegepant did not affect leukemic burden or araC sensitivity [50]. These authors therefore proposed that ADM, rather than CGRP, was the disease-promoting CALCRL ligand in AML.

Indeed, we also found that ADM protected human AML cell lines from chemotherapeutic drugs in vitro [74]. Nevertheless, although all of the primary AML samples we analyzed expressed the *CALCRL* and *RAMP1* mRNAs and upregulated them in response to treatment with araC, the majority did not express either *RAMP2* or RAMP3 [74]. Larrue et al. [50] found RAMP2 and RAMP3 protein expression in AML cell lines, but did not present any data from primary patient samples [50]. Also, normal hematopoietic cells, including HSCs, are well described to respond to CGRP, which is released from nociceptor nerves abounding in BM [54,62,69−73], making it plausible that CGRP −CALCRL signaling would also play a role in malignant hematopoiesis. To the best of our knowledge, similarly significant effects have not been described for ADM. On the other hand, human AML cell lines expressed ADM, but not CGRP, leading to the proposal of an autocrine mode of action of this CALCRL ligand [50]. Accordingly, only knockdown of *RAMP2*, but not of *RAMP1* or *RAMP3*, mimicked the effects of the *CALCRL* knockdown in AML cell lines [50], with the caveat that an effect of RAMP1 depletion might not be expected in the absence of CGRP. Another drawback of the proposed autocrine mechanism of action of ADM is that the reduction of leukemic burden during chemotherapy was associated with decreased ADM levels in PDX models [50], potentially indicating a diminution of the chemoprotective effect of ADM in the course of therapy. In contrast, the predominantly paracrine supply of CGRP [54,69,70,72] may represent a more plausible chemoprotective mechanism. Furthermore, olcegepant reduced leukemic burden and stem cell−related properties in a congenic AML mouse model [74]. On the other hand, no such effects were observed in the PDX model [50]. This discrepancy could be due to differences in the olcegepant doses used. Alternatively or additionally, because small molecule CGRP antagonists are species specific [75], there is a possibility that the effectiveness of olcegepant may be diminished in a heterologous system.

In summary, comprehensive evidence from several independent lines of research [48−50,74] suggests an important role for *CALCRL* in stemness and chemotherapy resistance of AML. The identification of the relevant ligand—CGRP, ADM, or both—requires further investigation, but small molecule inhibitors would be available, albeit at different stages of clinical development, in either case [55,56,76].

### Metastasis suppressor 1 (MTSS1)

In contrast to *SOCS2* and *CALCRL, MTSS1* was significantly downregulated at relapse of AML. *MTSS1* plays roles in cytoskeletal organization, signaling, and transcriptional regulation [77−81]. Suggesting a role as a tumor/metastasis suppressor, low *MTSS1* expression correlated with advanced stage and was an independent prognostic parameter for shorter survival in several solid tumor entities [82−86]. Moreover, *MTSS1* reduced proliferation and invasiveness of cancer cell lines [85,87,88]. *MTSS1* was also implicated in hematological malignancies: it was downregulated in human B-cell malignancies, and *Mtss1* knockout mice developed B-cell lymphomas [89]. In CML, reduced *MTSS1* expression was related to increased methylation of a CGI in its promoter [90]. Ectopic expression of the CML driver oncogene *Bcr−Abl* in murine hematopoietic cells effected downregulation of *Mtss1* in a manner partially sensitive to the ABL tyrosine kinase inhibitor imatinib [90]. Experimental re-expression of *Mtss1* in *Bcr-Abl*-expressing cells inhibited colony formation in semisolid media and decreased leukemic burden in recipient mice [90]. In AML, there was some evidence to suggest that methylation or low expression of *MTSS1* was associated with an unfavorable course of disease [91−93]. We therefore aimed to characterize in detail the expression and functional role of *MTSS1* in AML.

**Figure 4:**
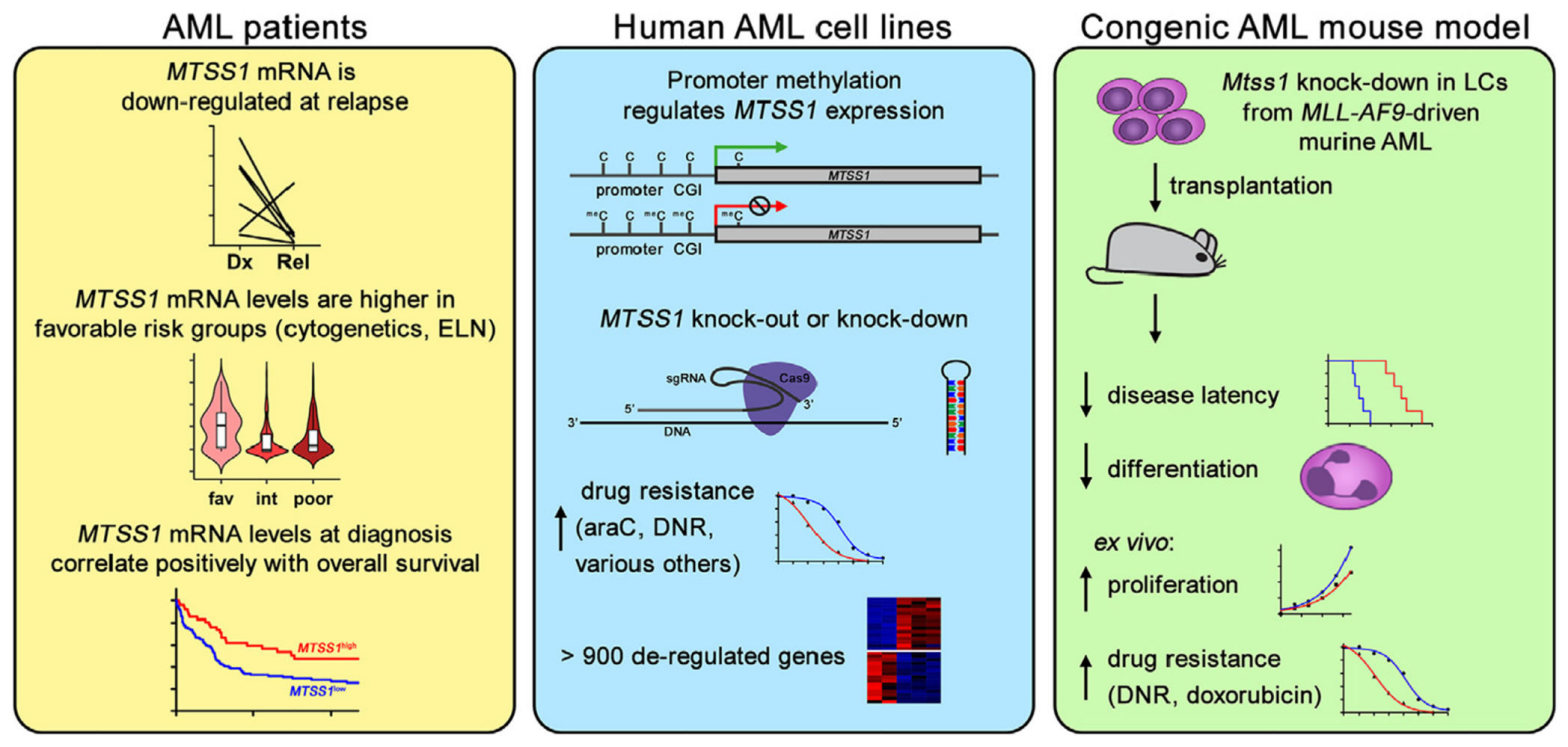
Downregulation of *MTSS1* is associated with relapse and poor outcome of AML, and experimental downregulation of *MTSS1* promotes AML progression and therapy resistance. *Cas9*=CRISPR associated 9; *DNR*=daunorubicin; *Dx*=diagnosis; *ELN*=European LeukemiaNet classification; *fav*=favorable; *int*=intermediate; *Rel*=relapse; *sgRNA*=single guide RNA.

In publicly available genomewide gene expression data, *MTSS1* was expressed at higher levels in AML patients with favorable risk according to cytogenetic or European LeukemiaNet classifications than in those with intermediate or poor risk. Moreover, low *MTSS1* transcript levels were associated with shorter overall survival in three independent patient cohorts [94]. qRT-PCR confirmed the downregulation of *MTSS1* at relapse of AML compared with paired diagnostic samples and healthy controls [94] (Figure 4). Together, the expression data from primary AML samples suggest a role for *MTSS1* downregulation in primary as well as acquired therapy resistance. The variable expression levels of *MTSS1* in human myeloid cell lines could be partially explained by a varying degree of methylation of the CGI in its promoter (Figure 4). Accordingly, the DNA methyltransferase inhibitor 5-aza-2′-deoxycytidine induced *MTSS1* expression in cell lines basally lacking it. Furthermore, *MTSS1* promoter methylation was moderately increased at relapse of AML as compared with matched diagnostic samples [94].

Supporting a contribution of *MTSS1* downregulation to chemotherapy resistance in AML, both CRISPR/ CRISPR-associated 9 (Cas9)-mediated knockout and shRNA-mediated knockdown of *MTSS1* in three different human myeloid cell lines increased their resistance to araC and daunorubicin, possibly via an increased DNA damage response [94]. To uncover possible therapeutic vulnerabilities of AML with low *MTSS1* expression, a robotic screen of more than 100 drugs under development or approved for oncological indications was performed. However, none of these substances exhibited increased effectivity toward *MTSS1* knockout cells. Rather, nine additional drugs representative of various classes, including, for example, the tyrosine kinase inhibitor regorafenib and the antimitotic vincristine, were less effective toward *MTSS1* knockout versus control cells [94] (Figure 4).

Genomewide gene expression profiling provided further evidence for central roles of *MTSS1* in normal and malignant myelopoiesis: 967 genes whose expression was significantly altered upon knockout of *MTSS1* in a human AML cell line were enriched for known targets of myeloid and/or leukemia-associated transcription factors, for example, MYC, RUNX1, CREB1, GATA2, STAT3, MEIS1, CEBPA, SPI1 (alias PU.1), and MECOM [94].

To validate the effect of reduced *Mtss1* expression on AML aggressiveness in vivo, an *MLL-AF9*-driven mouse model of AML was used. Short hairpin RNA-mediated knockdown of *Mtss1* in this model decreased disease latency to less than half. Furthermore, it strongly increased leukemic burden in BM and decreased the maturation of myeloid LCs. *Mtss1*-depleted cells also proliferated faster than control cells ex vivo and were more resistant to the anthracyclines daunorubicin and doxorubicin [94] (Figure 4).

In summary, downregulation of *MTSS1* in AML enhances disease progression and resistance both to conventional chemotherapy and to several targeted drugs.

### Lysine demethylase 6A (KDM6A)

**Figure 5:**
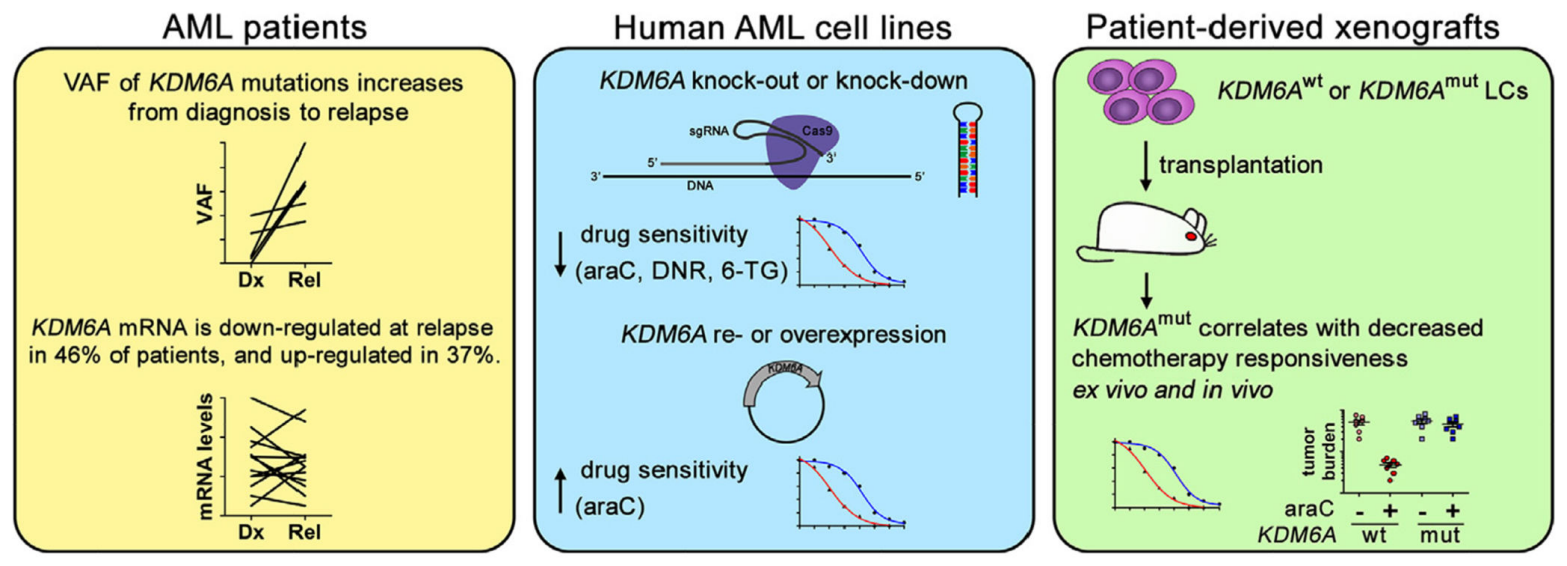
*KDM6A* can be mutated or downregulated at relapse of AML, and its inactivation decreases chemotherapy sensitivity. *araC*=cytosine arabinoside; *Cas9*=CRISPR-associated 9; *DNR*=daunorubicin; *Dx*=diagnosis; *mut*=mutated; *Rel*=relapse; *6-TG*=6-thioguanine; *sgRNA*=single guide RNA; *VAF*=variant allele frequency; *wt*=wild-type. In the xenograft model, ex vivo treatment was with araC and in vivo treatment with araC + liposomal daunorubicin.

Following a rationale related to ours, Stief et al. [95] identified *KDM6A* as downregulated at relapse of AML. The *KDM6A* gene is located on the X chromosome, encodes a histone H3 lysine 27 (H3K27)−specific demethylase, and carries somatic loss-of-function mutations in various types of cancer. *KDM6A* was also mutated in some patients with AML at the time of diagnosis, and the variant allele frequency of these mutations increased at relapse [95]. Downregulation of the *KDM6A* mRNA at relapse was observed in 46% of patients with AML, but 37% exhibited upregulation of this transcript [95] (Figure 5). Nevertheless, high DNA methylation levels of *KDM6A* at diagnosis were associated with shorter overall survival. Together, these data suggest a role for *KDM6A* mutation or downregulation in primary and relapse-associated therapy resistance. Indeed, in PDX from one patient each with wild-type and mutated *KDM6A*, the mutation was associated with increased resistance to araC ex vivo and to araC + liposomal daunorubicin in vivo [95] (Figure 5). In human AML cell lines, global H3K27 trimethylation levels were inversely correlated with KDM6A protein levels. Among male cell lines (which are hemizygous for *KDM6A*), those with *KDM6A* mutations were more resistant to araC. Knockdown or knockout of *KDM6A* in AML cell lines decreased their sensitivity to araC, and, albeit less consistently, to daunorubicin and 6-thioguanine. Conversely, re-expression or experimental overexpression of *KDM6A* increased araC responsiveness [95] (Figure 5). RNA-sequencing and inhibitor experiments suggested that the effects of KDM6A on araC sensitivity may be mediated via the nucleoside transporter protein SLC29A1 (alias ENT1) [95].

Taken together, mutation or downregulation of *KDM6A* may be another contributor to chemotherapy resistance, particularly toward the key drug araC, in AML. The consequences of *KDM6A* upregulation at relapse, observed in more than a third of patients, remain to be clarified.

## Conclusions and future perspectives

Relapse is frequent, generally associated with therapy resistance, and a major cause of death in AML. Molecular and genetic alterations promoting leukemia cell survival under therapy may either newly emerge or pre-exist in a subclone at diagnosis and are selected for during treatment. Changes that are newly acquired, or strongly enriched, at relapse are thus likely to contribute to therapy resistance and other properties of this disease stage. Certain mutations were recurrently gained at relapse of AML, but each of them was found only in a small proportion of patients [12,16]. AML can be caused by a variety of different driver mutations and, therefore, is genetically heterogeneous at diagnosis [8−10]. Conceivably, therapy resistance at relapse may likewise be affected by multiple and heterogeneous genetic alterations. However, investigations of the functional contributions of these mutations to chemotherapy resistance are largely pending.

In addition to genetic changes, changes in gene expression contribute to leukemogenesis, are of prognostic value, and may represent therapeutic targets in AML [3,8,13,31,96]. Interestingly, a study by Hackl et al. [22] suggested that changes in gene expression were more consistently acquired at relapse than genetic alterations, in that the transcript levels of numerous genes differed significantly between diagnosis and recurrence [22]. This study was based on a relatively small number of paired samples, but its results were validated through independent expression analyses, bioinformatics analyses, and functional studies. Nevertheless, investigation of larger patient cohorts and of patients with different cytogenetic characteristics at diagnosis will help to further advance our understanding of relevant gene expression changes at relapse. Indeed, Pabst et al. [29] reported related analyses; unfortunately, the corresponding Gene Expression Omnibus data sets do not include information about the disease stage of each sample.

The relapse signature established by Hackl et al. contained several genes previously implicated in key aspects of AML. Recent research additionally revealed roles for the deregulation of *SOCS2, CALCRL, MTSS1*, and *KDM6A* in therapy resistance and/or stemness of AML. CALCRL may be of particular interest, because small molecule inhibitors targeting it have already been developed [55,76].

Future studies should address the functional contributions of additional genes to relapse-related disease properties. Furthermore, single-cell expression analyses at the mRNA and protein levels will provide important insights into the heterogeneity of AML cell populations with respect to the expression of these genes. Gene expression patterns in AML LSCs, and their possible changes between diagnosis and relapse, will be of particular interest. For any resulting potential novel drugs, it will be pivotal to identify susceptible patient populations and to establish standardized procedures allowing their identification in clinical routine. Also, escape mechanisms will need to be examined. It is still early days in the investigation of molecular and genetic drivers of AML relapse. Much remains to be learned, but this research holds great promise to lead to the development of novel targeted drugs with the potential to improve outcome of a presently still often fatal malignancy.
